# Letter from Buena Vista, Stephenson County, Illinois

**Published:** 1854-03

**Authors:** Chas. G. Strohecker

**Affiliations:** Buena Vista, Stephenson Co., Ill.


☞ [We cheerfully give place to the following letter handed to us by Prof. Davis.]—Eds.

Buena Vista, Stephenson Co., Ill.
Feb. 27th, 1854.

Prof. Davis,—Dear Sir: While visiting your place, I wished to state a case to you that occurred in my practice, but your time being precious at that time, I did not wish to intrude upon it. I have therefore taken the liberty of communicating it to you from my case book. It was to me rather a singular case. I have mentioned it to several of my medical brethren, none of whom having met with anything of the kind. Should you consider it of sufficient importance, you may please hand it to the editor of the "Journal." I was called to see Mrs. M—— in labor with her third child, August 25th, 1853, at 1 A. M. When I arrived I found her in a severe pain. I immediately made an examination, and found the head rising under the pubes. The next pain brought a very small male child. In making manipulation over the abdomen, I found it remained as large as before the expulsion of the child. In about fifteen minutes slight pains came on; I introduced my finger into the vagina, found the placenta there, (which was also very small). I removed that, and searched for the os, which was high up and contracted. In a very short time the pains came on again, *regular* and *strong*; with intervals of about ten minutes between each. The abdomen remaining so large, and the pains so regular, I pronounced it a "twin case." There being no untoward symptoms to demand haste, I thought I would leave nature to take its course. Thus I waited two hours and a half; there being no alteration in the size or position of the os, I began to think something else must be done in the case—the pains all the while keeping regular, but of shorter duration.

I placed her in the position for introducing the hand; after anointing my hand, I introduced it into the vagina. It was with considerable difficulty I introduced the two first fingers through the os, when there was an explosion, long and loud, so loud that it might have been heard in any part of the house, and to my surprise and mortification, I found my second child ended in *gas*. The abdomen instantly became relaxed, the uterus contracted down, and remained contracted. No hæmorrhage or after pains followed, and the woman made a rapid recovery.

Very respectfully yours, &c.,

CHAS. G. STROHECKER.

